# Hospital solid waste management strategies to prevent healthcare-associated infections from occupational exposure to bloodborne pathogens and improve occupational safety

**DOI:** 10.3389/fpubh.2025.1499463

**Published:** 2025-01-29

**Authors:** Augusto Jorge Antonio Ibáñez-Cruz, Alejandra Micaela Elena Vergara-Florián, William C. Algoner

**Affiliations:** Carrera de Ingeniería Biomédica, Facultad de Ingeniería, Universidad Tecnológica del Perú, Lima, Peru

**Keywords:** nosocomial infections, hospital solid waste, occupational safety, waste management, health training

## Abstract

Improving occupational safety and public health is crucial to reducing nosocomial infections in healthcare workers due to the ongoing deficiencies in solid waste management in private clinics. For this reason, it is necessary to implement appropriate solid waste management practices to mitigate risks for clinical staff, patients, and the environment. This research focused on reducing the risk of nosocomial infections in healthcare workers exposed to bloodborne pathogens. It was carried out at the private clinic and concentrated on properly managing hospital solid waste, with special attention to occupational health and safety. 400 health workers were trained online during six sessions, addressing biosafety, conditioning, segregation, storage, collection, and transportation of solid waste. The amount of waste produced in kilograms daily was 232.76 bio-contaminated, 11.23 special, and 218.58 joint. Bio-contaminated waste included patient care, bags with human blood and blood products, surgical and anatomicalpathological waste, sharps and biological objects, pharmaceuticals, and hazardous and radioactive chemicals. Proper solid waste management, supported by adequate training, contributed to a significant decrease in the incidence of nosocomial infections: two cases were reported in August and one in September, and there was no incidence of cases from October to December. The estimation of the method used for solid waste disposal showed an acceptable degree in the stages of conditioning, segregation, primary storage, internal transport within the clinic, and central storage. In addition, the occupational health and safety of the personnel at the private clinic was improved.

## 1 Introduction

Reducing the risk of nosocomial infections is a critical challenge for occupational safety and wellness in healthcare settings. These infections, also known as healthcare-associated infections (HAIs), are acquired by patients during their stay in a hospital or other healthcare facility and were not present or incubating at the time of admission; they can cause severe complications for patients and healthcare personnel, increase morbidity, mortality, prolong the duration of hospitalization, and increase healthcare costs (1, 2).

Hospital waste management, the safe and efficient handling of waste produced in healthcare facilities,^1^ is crucial in preventing HAIs. The World Health Organization warns that poorly managed hospital waste can be a source of pathogens that contribute to the spread of nosocomial infections. Therefore, implementing proper waste handling practices, including segregation, storage, transportation, and disposal, is essential to ensure a safe hospital environment (1–4).

Recent studies have shown that effective hospital waste management decreases the incidence of HAIs and optimizes working environments for healthcare personnel, reducing exposure to biological and chemical hazards (4). In addition, proper waste disposal and treatment contribute to environmental sustainability, an increasingly notable element in hospital management (2, 5, 6).

A study demonstrated that implementing strict waste management protocols in hospital facilities significantly decreased nosocomial infections (4). In addition, continuous training of healthcare personnel in waste management and providing adequate resources are crucial factors for the success of these strategies (2, 6–8).

Proper evaluation of the procedures for handling solid hazardous hospital waste will help minimize its environmental impact by implementing a plan that controls the most difficult sites for handling these wastes. Therefore, the efficiency with which this is carried out and the stages designed for such purposes are fulfilled will have an important impact both internally and externally (8).

Private clinic, like many institutions, experienced increased nosocomial infections due to solid waste management deficiencies during 2021–2024. This study aims to evaluate the effectiveness of a training program for healthcare personnel to optimize waste management and reduce nosocomial infections, which is in line with current legislation in the country on solid waste management in healthcare facilities (2).

Likewise, the research was justified because proper hospital waste management will help avoid harmful effects and health risks for clinical staff, patients, visitors, and the environment.

### 1.1 Climate and waste impacts on public health

Ineffective waste management not only has direct implications on human health, but also on the environment. The World Health Organization (WHO) and other international bodies have warned that poorly managed medical waste can contribute significantly to climate change and environmental problems, such as water and soil contamination. In addition, recent statistics show that poor management of hospital waste can lead to infectious disease and epidemic outbreaks in vulnerable communities. For example, a recent study highlights that developing regions experience a 30% increase in diseases related to inadequate medical waste disposal (7).

### 1.2 Infections of occupational origin

Occupational infections represent another critical component of public health. Healthcare workers face significant risks due to exposure to blood-borne pathogens, such as HIV and hepatitis B and C. A 2022 report highlights that 58% of occupational exposures to pathogens occur due to improper handling of contaminated waste (9). This underscores the need for more stringent occupational safety measures and the implementation of up-to-date preventive practices.

## 2 Materials and methods

### 2.1 Effective solid hazardous waste management strategies

#### 2.1.1 Selection of the study sample

The study sample included 400 health professionals from the private clinic who participated in the training and sensitization program, which was the focus of the research. These professionals were selected because they actively participate in hospital activities and direct interaction with solid waste management processes. Including this sample allowed a comprehensive evaluation of the impact of the training on daily practice, its effects on safety and health, and the management of in-hospital waste. Proper waste management can drastically reduce the risk of nosocomial infections, safeguard staff health, and ensure a safer hospital environment (2).

#### 2.1.2 Data collection procedure permitting and coordination

The director of the private clinic authorized the conduct of observed data on how solid waste is managed. This initial step was crucial to ensure the access and collaboration of all personnel involved. Authorization involved submitting a detailed plan for the research, including objectives, methods, and proposed activities. Once approval was obtained, reconciliation with the medical and administrative staff took place to establish the methodology and specific activities of the study. This coordination included scheduling observations and organizing briefings for those involved in the research (2).

#### 2.1.3 Direct observation

Direct observations were made of the cleaning routes and schedules in different clinic areas. These were carried out as follows:

- Cleaning Routes: The routes established for the collection and transportation of solid waste from the different units of the hospital to the storage and final disposal points were observed. This made it possible to identify possible inefficiencies and risks in waste management.
- Cleaning schedules: The schedules for cleaning and waste management activities in the different areas of the hospital were recorded. This analysis helped to determine the effectiveness of the established schedules and their impact on reducing the risk of nosocomial infections.
- Frequency of cleaning: The frequency of cleaning and waste management was observed, evaluating whether the time intervals were adequate to minimize the risk of accumulation of waste and the spread of infections.

Compliance with Protocols: During the observations, compliance with the protocols established for the safe management of solid hospital waste, including segregation, the use of personal protective equipment, and collection and transportation techniques, was verified.

These observations were fundamental for obtaining a clear and detailed view of current waste management practices at private clinic. This made it possible to identify areas for improvement and evaluate the training’s impact on reducing the risk of nosocomial infections and on the occupational health and safety of the personnel.

#### 2.1.4 Waste production

Daily solid waste production was estimated using 2021 Solid Waste Management Program data. Waste was classified as biocontaminated, unique, and standard. This classification made it possible to identify the different categories of waste and establish specific plans for their safe management and disposal. The estimate was made by analyzing waste generation records, facilitating the evaluation of current practices, and identifying critical areas requiring improvement.

#### 2.1.5 Identification of risk points

The services and areas that generate biocontaminated and waste were identified, highlighting those with the highest risk of nosocomial infections. This identification was made through a detailed analysis of hospital areas, including operating rooms, intensive care units, laboratories, adult emergency, X-ray, isolation areas, maintenance workshops, and diagnostic imaging units. The objective was to focus training and improvement efforts on the areas at most significant risk, thus ensuring a more effective and targeted intervention.

### 2.2 Development and implementation of training and awareness programs

#### 2.2.1 Implementation of the training and awareness program

After identifying the point of risk, a virtual training program was designed and implemented, consisting of six 1-h sessions. This program included biosafety and solid waste management topics to educate and sensitize personnel on best practices and procedures for safe waste management. The training sessions were developed and recorded using online education platforms, allowing all those involved in the study to participate and access the content broadly (2). The proposal sought to train health professionals at private clinic in the correct management of solid waste to prevent health risks and promote environmental quality, as well as the health and well-being of people, in compliance with current legislation on waste management in health care centers. In this sense, six virtual trainings were carried out in one-hour sessions, whose subject matter covered the following topics (Table 1).

**Table 1:** Training and awareness program.

session	date	theme	specific content
1	August 21	Solid Waste and Biosafety	Classification of waste Generation and composition of waste Environmental impact of solid waste Basic concepts of biosafety Risks associated with improper waste handling
2	September 4	Conditioning of Solid Waste	Waste packaging Containment and packaging of waste according to classification Proper use of personal protective equipment (PPE)
3	September 18	Segregation of Solid Waste	Waste segregation criteria Identification of different types of waste (organic, recyclable, hazardous) Importance of segregation at the source
4	October 2	Primary and Intermediate Storage	Design and characteristics of storage sites Collection frequency Safety measures in primary and intermediate storage
5	October 16	Internal Collection and Transport	Waste collection routes Equipment and vehicles used for internal transport Safety measures during waste transport
6	October 30	Central or Final Storage	Final disposal of waste Treatment options: reduction, reuse, and recycling Environmental regulations applicable to waste disposal

Each session was organized in a methodological sequence that began with activating previous knowledge, then generating new learning and applying this knowledge in daily life. The evaluation was based on understanding the concepts and regulations presented during the sessions. These virtual trainings were essential to ensure that participants were well informed and prepared to manage solid waste safely and in compliance with current legislation, thus contributing to the prevention of health risks and the protection of the environment.

#### 2.2.2 Evaluation of the training and awareness program

After the training, unannounced visits were made to evaluate the performance of the solid waste management procedures. During these visits, adherence to the established protocols was observed, and data on reducing nosocomial infections was collected. The data obtained were compared with the records before the implementation of the training plan, allowing a quantitative evaluation of the impact of the interventions carried out. This evaluation included analysis of infection rates, compliance with biosafety practices, and staff satisfaction with the training program.

This systematic approach to data collection and analysis provided a reasonable judgment of hospital solid waste management in reducing the risk of nosocomial infections and its impact on occupational health and safety at the private clinic.

## 3 Results

### 3.1 Daily generation of solid hospital waste at the private clinic

The daily estimate of biocontaminated waste generated at the private clinic was 232.76 kilograms. The floors that contributed most to this production were floor 1, with 77.61 kilograms, and floor 6, with 21.85 kilograms. This waste includes patient care, blood bags, and surgical and biological waste. In addition, daily production of 11.23 kilograms of waste was estimated, with floor 4, 2.25 kilograms, and floor 1, 2.03 kilograms, generating the most waste. These wastes include pharmaceutical, hazardous chemical, and radioactive wastes. The generation of everyday waste, that is, waste without direct contact with patients, was 218.58 kilograms (Table 2). These materials are not considered hazardous and do not require unique management in terms of biosafety (2).

**Table 2:** Estimated daily production of solid waste according to services/areas.

Floors	Services/Areas	Biocontaminated (kg.)	Special (kg.)	Common (kg.)
Basement 3	Construction storage	2	1.1	24.9
	Pharmacy warehouse			
	Hospital pharmacy			
	Canteen			
	Maintenance workshop			
	Logistics warehouse			
	Cleaning warehouse			
	Cleaning office			
	Diluter rooms			
	Archive			
Basement 2	General services	12.85	0.25	25.1
	Sterilization center			
	Nutrition and diets			
	Medical fees			
	Cashier’s office—vault			
	Human resources			
	Medical audit			
	Production chain			
	Clinical laboratory			
	Mortuary			
	Monitoring center			
Basement 1	Tomography	20.6	0.15	20.95
	Bone densitometry			
	Mammography			
	X-ray			
	Accounting			
	Treasury			
	Systems			
	Surgical Center (dressing rooms)			
	Blood Bank			
	Maintenance Office			
	Commercial Office			
	Laboratory—sampling			
	Physical medicine and rehabilitation			
	Ultrasound	77	2.03	24.7
Floor 1	Surgical Center			
	Adult emergency			
	X-ray			
	Emergency admission			
	Central Pharmacy			
Floor 2	Hospitalization—2nd floor	19.3	0.7	19.03
	Pediatric emergency			
	EPPS Warehouse			
	Ambulatory admission			
	Hospital admission			
	Emergency admission			
	User service platform			
Floor 3	Hospitalization—3rd Floor	6.55	1.5	13
	3rd Floor Offices			
	Topic			
	Epidemiology Office			
	Medical Subdirection			
	Ambulatory Admission			
	Budgets			
Floor 4	Ambulatory Admission	19.35	2.25	16.85
	Family plan			
	Tele-consultation			
	Hospitalization—4th Floor			
	4th Floor Consultation Rooms			
Floor 5	Hospitalization—5th Floor	16.9	0.9	18.8
	5th Floor Offices			
	Endoscopic Center			
	Outpatient Admission			
	Documentary procedure			
	Family plan			
	Nurse’s office			
	Occupational Health			
	Traumatology topic			
Floor 6	Call center	21.85	0.5	24.6
	Process office			
	Ambulatory admission			
	General Management			
	Administrative Management			
	6th floor office			
	Intensive Care Unit			
	Topical			
Floor 7	Hospitalization—7th Floor	16.51	1.6	12.9
Floor 8	Hospitalization—8th Floor	19.85	0.25	17.75
Total		232.76	11.23	218.58

### 3.2 Areas that generate biocontaminated and special wastes

The results reveal that the surgical area, adult emergency room, radiology department, and intensive care areas are the principal creators of biocontaminated waste. Pharmaceutical waste occurs mainly on floor 4, while hazardous chemical and radioactive waste is identified in specific areas such as the maintenance shop and diagnostic imaging units (2).

#### 3.2.1 Biocontaminated waste

Surgical Center: The surgical center is one area that generates the most biocontaminated waste due to the high frequency of invasive procedures. These procedures create a variety of waste, including single-use materials such as gloves, masks, gowns, gauze, and other surgical instruments contaminated with blood and body fluids. Implementing strict protocols for segregating, storing, and disposing of these wastes is crucial to avoid risks of infection and cross-contamination.

Adult Emergency: The adult emergency generates a considerable amount of biocontaminated waste due to the high influx of patients with various medical conditions, from trauma to infectious diseases. The people who work here must be prepared to handle this waste, using appropriate containers and following disinfection and disposal protocols to minimize risks.

X-ray: Although X-ray departments generate less biocontaminated waste than surgical areas, they still produce waste, such as gloves, gowns, and other materials used during diagnostic procedures. Proper waste management is critical to prevent the spread of biological contaminants.

Intensive Care Units (ICUs): ICUs are critical areas where patients with severe conditions and infectious diseases are managed, resulting in a high generation of biocontaminated waste. These include medical equipment waste, life-supporting materials, and drug waste. Proper management of these wastes is essential to maintain a safe environment for all people in this department, whether they are patients or workers.

#### 3.2.2 Special waste—pharmaceutical waste

On floor 4, a high generation of pharmaceutical waste was identified due to administering and disposing of expired medicines, leftover doses, and waste from pharmaceutical preparations. It is crucial to have an efficient system for segregating and disposing of pharmaceutical waste, following local and international regulations to prevent environmental contamination and public health risks.

#### 3.2.3 Hazardous chemical and radioactive waste

The maintenance shop generates hazardous chemical waste, such as solvents, oils, paints, and other chemical products used in maintaining equipment and facilities. These wastes must be managed following specific protocols for their segregation, storage, and final disposal, minimizing the risks of exposure and environmental contamination.

Diagnostic imaging units generate radioactive waste, especially in procedures that use radioactive isotopes and other materials for diagnosis and treatment. These wastes must be managed under strict radiation safety regulations, ensuring their correct handling from segregation to disposal to protect both personnel and the environment (Table 3).

**Table 3:** List of areas that produce biocontaminated and special wastes.

Floors	Area	Class	Description
Basement 3	Pharmacy Warehouse	Class A: Biocontaminated Waste	A1: Patient Care Waste
	Hospital Pharmacy	Class A: Biocontaminated Waste	A1: Patient Care Waste A5: Sharps
		Class B: Special Wastes	B1: Pharmaceutical Waste
	Maintenance Shop	Class A: Biocontaminated Waste	A1: Patient Care Waste
		Class B: Special Wastes	B1: Hazardous Chemical Waste
Basement 2	General Services	B1: Hazardous chemical wastes	A1: Patient Care Waste
	Central Sterilization Plant	Class A: Biocontaminated Waste	A1: Patient Care Waste
		Class B: Special Wastes	A2: Biological waste A4: Surgical and anatomo-pathological wastes A5: Sharps
	Nutrition and Diets	Class A: Biocontaminated Waste	A1: Patient Care Waste
	Clinical Laboratory	Class A: Biocontaminated Waste	A2: Biological waste A3: Bags containing human blood and blood derivatives A5: Sharps
		Class B: Special Wastes	B1: Hazardous chemical waste
	Mortuary	Class A: Biocontaminated Waste	A1: Patient care waste A3: Bags containing human blood and blood derivatives
Basement 1	Tomography	Class A: Biocontaminated Waste	A1: Patient Care Waste
		Class B: Special Wastes	B2: Pharmaceutical waste B3: Radioactive waste
	Bone Densitometry	Class A: Biocontaminated Waste	A1: Patient Care Waste
		Class B: Special Wastes	B3: Radioactive waste
	Mammography	Class A: Biocontaminated Waste	A1: Patient Care Waste
		Class B: Special Wastes	B3: Radioactive waste
	X-Ray	Class A: Biocontaminated Waste	A1: Patient Care Waste
		Class B: Special Wastes	B3: Radioactive waste
	Surgical Center (Dressing rooms)	Class A: Biocontaminated Waste	A1: Patient Care Waste
	Laboratory-Sampling	Class A: Biocontaminated Waste	A1: Patient Care Waste A2: Biological waste A3: Bags containing human blood and blood derivatives A5: Sharps
	Physical Medicine and Rehabilitation	Class A: Biocontaminated Waste	A1: Patient Care Waste
	Ultrasound	Class A: Biocontaminated Waste	A1: Patient Care Waste
Floor 2	Hospitalization 2nd floor	Class A: Biocontaminated Waste	A1: Patient care waste A3: Bags containing human blood and blood derivatives A4: Surgical and anatomo-pathological waste A5: Sharps
		Class B: Special Wastes	B2: Pharmaceutical Waste
	Pediatric Emergency	Class A: Biocontaminated Waste	A1: Patient care waste A3: Bags containing human blood and blood products A5: Sharps
		Class B: Special Wastes	B2: Pharmaceutical waste
	Ambulatory Admission Hospital Admission	Class A: Biocontaminated Waste	A1: Patient Care Waste
		Class B: Special Wastes	B3: Radioactive wastes
	Emergency Admission	Class A: Biocontaminated Waste	A1: Patient Care Waste
	User Care Platform	Class A: Biocontaminated Waste	A1: Patient Care Waste
		Class B: Special Wastes	B2: Pharmaceutical Waste
Floor 3	Hospitalization 3rd floor	Class A: Biocontaminated Waste	A1: Patient care waste A2: Biological waste A3: Bags containing human blood and blood products A5: Sharps
		Class B: Special Wastes	B2: Pharmaceutical Waste
	3rd floor offices	Class A: Biocontaminated Waste	A1: Residuos de atención al paciente A3: Bolsas conteniendo sangre humana y hemoderivados A5: Punzocortantes
		Class B: Special Wastes	B2: Pharmaceutical Waste
	Topic	Class A: Biocontaminated Waste	A1: Patient care waste A3: Bags containing human blood and blood derivatives A5: Sharps
		Class B: Special Wastes	B2: Pharmaceutical Waste
	Ambulatory Admission	Class A: Biocontaminated Waste	A1: Patient Care Waste
	Budgets	Class A: Biocontaminated Waste	A1: Patient Care Waste
Floor 4	Ambulatory Admission	Class A: Biocontaminated Waste	A1: Patient Care Waste
	Hospitalization 4th floor	Class A: Biocontaminated Waste	A1: Patient care waste A2: Biological waste A3: Bags containing human blood and blood products A5: Sharps
		Class B: Special Wastes	B2: Pharmaceutical Waste
	4th floor offices	Class A: Biocontaminated Waste	A1: Patient care waste A3: Bags containing human blood and blood derivatives A5: Sharps
		Class B: Special Wastes	B2: Pharmaceutical Waste
Floor 5	Hospitalization 5th floor	Class A: Biocontaminated Waste	A1: Patient care waste A2: Biological waste A3: Bags containing human blood and blood products A5: Sharps
		Class B: Special Wastes	B2: Pharmaceutical Waste
	5th floor offices	Class A: Biocontaminated Waste	A1: Patient care waste A3: Bags containing human blood and blood derivatives A5: Sharps
		Class B: Special Wastes	B2: Pharmaceutical Waste
	Endoscopic Center	Class A: Biocontaminated Waste	A1: Patient Care Waste A5: Sharps
		Clase B: Residuos Especiales	B1: Hazardous Chemical Waste B2: Pharmaceutical Waste
	Ambulatory Admission	Class A: Biocontaminated Waste	A1: Patient Care Waste
	Documentary Procedures	Class A: Biocontaminated Waste	A1: Patient Care Waste
	Topical Traumatology	Class A: Biocontaminated Waste	A1: Patient Care Waste A5: Sharps
Floor 6	Ambulatory Admission	Class A: Biocontaminated Waste	A1: Patient Care Waste
	6th Floor Office	Class A: Biocontaminated Waste	A1: Patient care waste A3: Bags containing human blood and blood derivatives A5: Sharps
		Class B: Special Wastes	B2: Pharmaceutical Waste
	Intensive Care Unit	Class A: Biocontaminated Waste	A1: Patient care waste A2: Biological waste A3: Bags containing human blood and blood products A5: Sharps
		Class B: Special Wastes	B2: Pharmaceutical Waste
	Topic	Class A: Biocontaminated Waste	A1: Patient Care Waste A5: Sharps
		Class B: Special Wastes	B2: Pharmaceutical Waste
Floor 7	Hospitalization 7th floor	Class A: Biocontaminated Waste	A1: Patient care waste A2: Biological waste A3: Bags containing human blood and blood products A5: Sharps
		Class B: Special Wastes	B2: Pharmaceutical Waste
Floor 8	Hospitalization 8th floor	Class A: Biocontaminated Waste	A1: Patient care waste A2: Biological waste A3: Bags containing human blood and blood products A5: Sharps
		Class B: Special Wastes	B2: Pharmaceutical Waste

### 3.3 Evaluation of solid waste treatment

**Figure 1:**
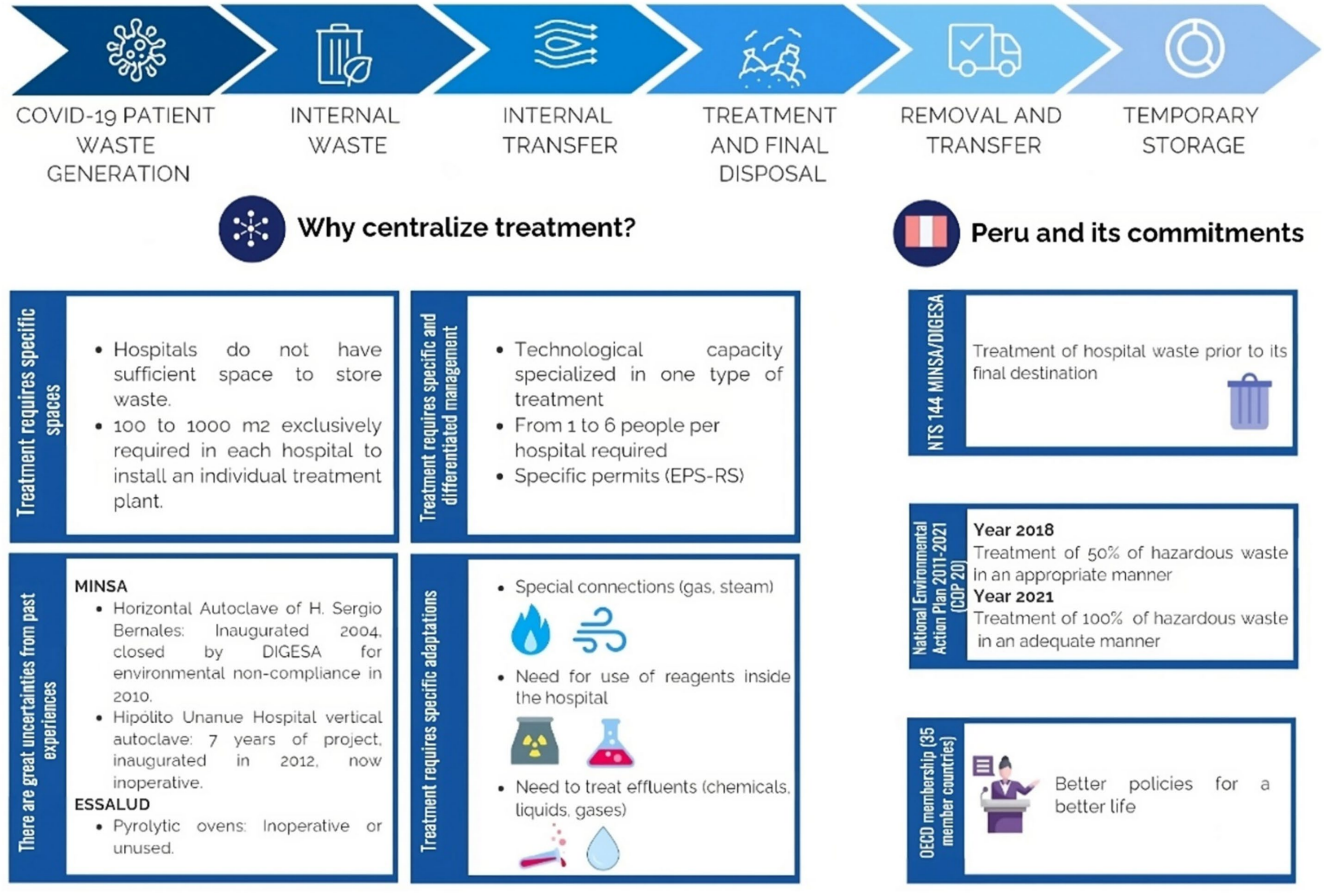
Safe management of waste originating in hospitals during patient care for COVD-19 virus (Biopathogenic Waste).

The evaluation of solid waste treatment at the private clinic included several critical stages, each subjected to detailed analysis to determine the effectiveness and compliance with the protocols implemented (Figure 1).

#### 3.3.1 Conditioning

In this phase, the services and areas were organized and equipped with materials such as bags, containers, buckets, and rigid containers appropriate for the different categories of waste generated at the clinic. During the unannounced visits, it was observed that all clinic areas were adequately equipped with the necessary materials, facilitating the correct segregation from where the waste is produced. The cleaning and maintenance personnel showed adequate knowledge of the importance of using these supplies and how to distribute them in the different areas.

#### 3.3.2 Segregation and primary storage

During this phase, waste was sorted at the place of origin, placing it in the appropriate container according to its type. It was noted that the clinical and administrative staff complied with the established guidelines for waste separation. All types of waste were correctly deposited in the assigned containers, thus reducing the risk of cross-contamination. The training received by the staff was essential to ensure the proper execution of this stage, and a significant improvement in segregation practice was noted compared to data before the implementation of the plan.

#### 3.3.3 Intermediate storage

Intermediate storage involves temporarily collecting waste in places or environments designated explicitly for this purpose. These places were strategically distributed within the different areas of the clinic to facilitate the management and control of the waste until its total elimination. This strategy optimized the collection process and minimized the risks of contamination and improved efficiency in the integral management of clinical waste.

#### 3.3.4 Collection and internal transportation

The transfer of waste within the clinic was carried out by personnel with the appropriate protective equipment and safety tools. Appropriate vehicles were used to differentiate the collection of different types of waste. During the evaluation, it was observed that the transportation personnel followed the protocols rigorously, minimizing the danger of infection by pathogens. Implementing well-defined collection routes and using appropriate vehicles ensured efficient and safe transport within the clinic.

#### 3.3.5 Final storage

In this phase, waste generated at the source or during primary storage was temporarily placed in a suitable space for subsequent treatment or final disposal. Inspections revealed that the final storage areas were properly conditioned, following safety and infection prevention protocols. These areas had adequate ventilation, clear signage, and specific containers for each type of waste, facilitating their safe handling.

#### 3.3.6 Valuation (optional)

In waste valorization, operations were carried out to reuse the remains or some of the elements of which they are composed. This valorization allowed these materials to be helpful, replacing other resources in the production processes. Two types of valorizations were ensured: material, where waste was transformed into new products or raw materials, and energetic, where it was converted into energy sources. This practice contributed to reducing environmental impact and optimized the use of available resources, promoting more sustainable and efficient waste management within the clinic.

#### 3.3.7 Treatment

Solid waste management encompasses implementing various processes, methods, and techniques to alter the waste’s physical, chemical, and biological properties. These alterations aimed to minimize or eradicate the waste’s risk to health and the environment. This was achieved by adequately preparing the waste for future valorization or final disposal, ensuring that the materials were safely reused efficiently or disposed of. Valorization made converting waste into new materials or energy sources possible, contributing to environmental sustainability. At the same time, final disposal ensured compliance with all current sanitary and environmental regulations, underscoring the clinic’s commitment to environmental safety and public health.

#### 3.3.8 Collection and external transportation

Solid waste treatment was carried out, which included waste collection by an operating company duly registered with the competent authority. This process covered from the health facility and the authorized medical service to the research center, culminating in the final disposal of the waste. Vehicles authorized by the municipality and the Ministry of Transportation and Communications were used for transportation, ensuring that the hazardous waste was not mixed with municipal waste. All of this was done in compliance with current sanitary and environmental regulations to minimize risks and ensure proper management of the waste generated at the clinic.

#### 3.3.9 Final provision

At the clinic, specific processes and operations were carried out to treat and dispose waste in designated sites. This last step in waste management was carried out on an ongoing basis, ensuring that sanitary and environmentally safe conditions were maintained. These procedures ensured that waste was appropriately managed, minimizing risks to the population’s well-being and the environment and complying with all current waste management regulations.

In the nine levels evaluated, it was observed that the clinic’s personnel showed a satisfactory degree of adherence to the procedures established for solid waste management. These results are supported by a notable decrease in nosocomial infections, underscoring the interventions’ effectiveness (Table 4). Ongoing training and strict adherence to waste management protocols not only improved the daily practices of the staff but also contributed to establishing a safer and healthier work environment.

**Table 4:** Solid waste treatment by stages.

Stages	Assessment criterion	Indicator	Valorization	Justification
Conditioning	Adequacy of pretreatment	% of waste properly conditioned	Acceptable	95% of the containers are correctly identified according to waste types.
Segregation and Primary Storage	Compliance with safety standards	% of contaminated containers; compliance with labels	Acceptable	Less than 2% of the containers are cross contaminated. 100% of the containers have clear identification labels.
Intermediate Storage (COVID-19)	Storage conditions	Temperature, humidity, ventilation; compliance with specific protocols; % of routes followed; daily frequency; use of personal protective equipment.	Acceptable	Proper storage conditions are maintained for COVID-19 waste, according to established protocols. No incidents have been reported.
Collection and Internal Transportation	Compliance with routes and frequencies; transport safety	% of routes complied with; daily frequency; use of personal protective equipment	Acceptable	100% of the routes are being followed as scheduled. Adequate personal protective equipment is used. No incidents have been reported during transport.
Final Storage	Storage conditions	Area security; access log	Acceptable	The final storage area is equipped with adequate security measures (fencing, signage). Access to the area is recorded.
Valorization (Optional)	Recovery potential	% of waste valorized; type of valorization; % removal of pathogens; compliance with labels	Acceptable	Seventy percent of the organic waste is recovered through composting.
Treatment	Treatment efficiency	% of pathogen elimination; compliance with regulations	Acceptable	Treatment guarantees the elimination of 99% of pathogens. Current regulations for the treatment of hospital waste are complied with.
External Collection and Transport	Regulatory compliance; traceability	Transportation documentation; record of final destination	Acceptable	All the documentation required for transportation is complete. There is a record of the final destination of the waste.
Final Disposal	Regulatory compliance	% of waste correctly disposed of; documentation	Acceptable	All waste is disposed of in an authorized sanitary landfill. There is documentation to support final disposal.

**Figure 2:**
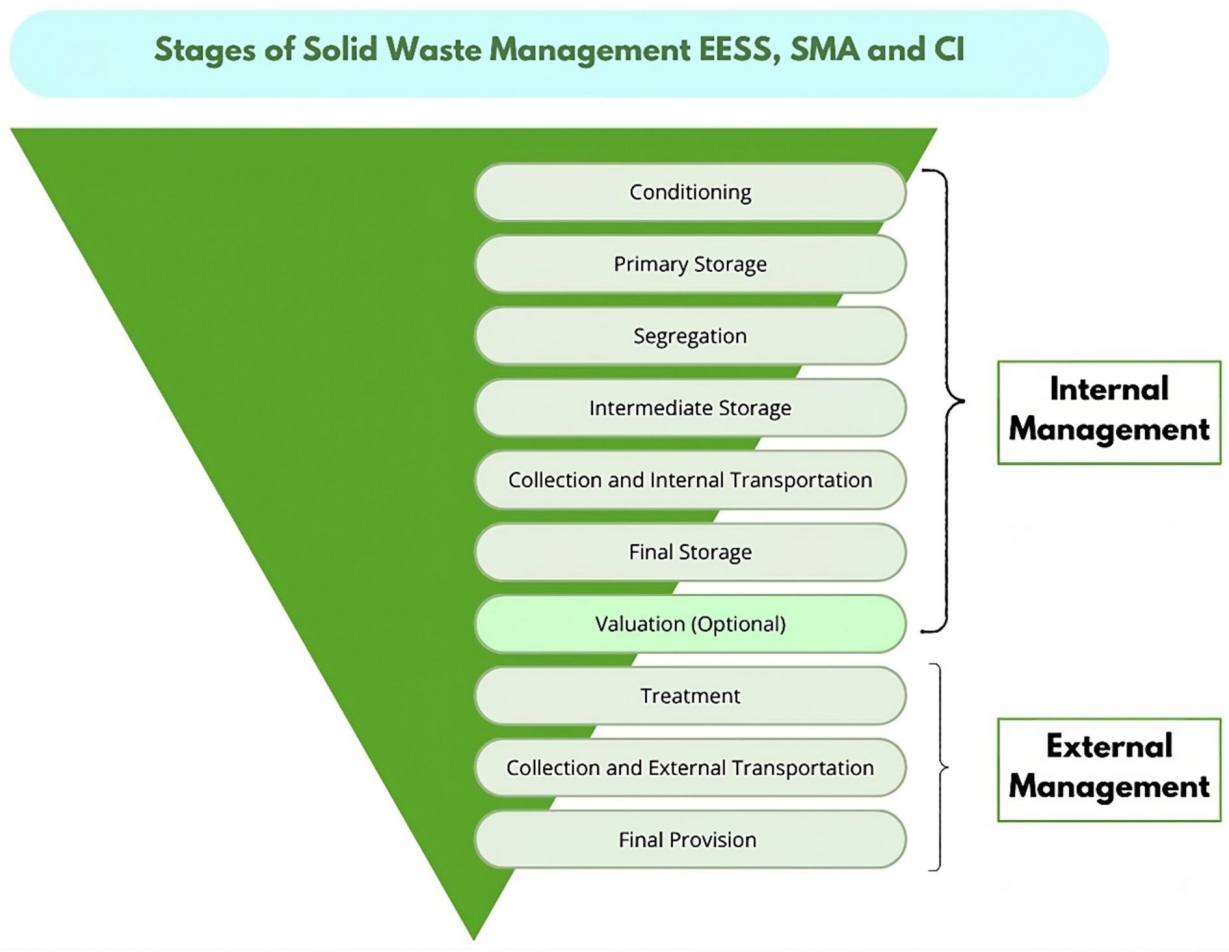
Solid waste management in the context of the national vaccination plan against COVID-19-2022 (14).

The success of this plan demonstrates the importance of a comprehensive approach to solid waste management that goes beyond staff training to include adequate conditioning of areas, proper segregation and storage, and efficient and safe internal transportation. Private clinic experience can serve as a model for organizations wishing to optimize their waste management procedures and reduce the risk of nosocomial infections (Figure 2).

The Table 4 provides a comprehensive view of how the hospital waste supply chain is managed, revealing several key indicators demonstrating the effectiveness of the implemented management practices. The encouraging results suggest that current strategies lead to efficient and sustainable hospital waste management.

### 3.4 Training and awareness raising results

Between August and October 2024, a training and awareness program on solid waste management and biosafety was implemented for 400 sanitation workers. The program consisted of six sessions, each focusing on a specific topic for improving waste handling practices and safety. The results obtained were as follows:

In the first session of the study, which focused on solid waste and biosafety, significant results were observed in workers’ understanding and handling.

Initially, 65% of the workers had a basic knowledge of waste classification. However, by the end of the training, 95% of the workers clearly understood the classification, how waste is generated and composed, and the basic concepts of biosafety. In addition, there was a marked improvement in waste management practices, evidenced by a 20% decrease in incidents related to proper waste handling and disposal.

Workers made notable improvements in the second session of the study, which focused on solid waste preparation. Initially, 60% of the workers were familiar with basic packaging techniques and personal protective equipment. After training, 92% of the workers demonstrated improved skills in packaging, containment, and correct use of PPE. In addition, there was a 30% increase in the proper use of PPE during waste handling, indicating a significant improvement in safety practices.

Significant results were obtained from the workers in the third session of the study, which was dedicated to solid waste segregation. Initially, 55% of the workers understood the essential criteria for waste segregation. At the end of the training, 90% of the workers could correctly identify and segregate organic, recyclable, and hazardous waste. In addition, there was a 25% increase in the correct waste separation, reflecting a considerable improvement in waste management.

The workers recorded substantial improvements in the fourth training session, which focused on primary and intermediate waste storage. In the beginning, 50% of the workers were aware of the design and characteristics of the storage sites. After training, 88% of workers clearly understood waste storage collection frequency and safety measures. There was also a 15% improvement in the organization and safety of intermediate storage sites, indicating significant progress in waste management.

Significant improvements were observed in the fifth session of the study, which focused on internal waste collection and transportation. Initially, 45% of the workers knew the routes and safety measures during waste transportation. At the end of the training, 85% of the workers demonstrated skills in route planning and the safe use of equipment and vehicles. In addition, there was a 10% reduction in incidents during internal waste transportation, improving safe handling practices.

Significant progress was made in the sixth session of the study, which was dedicated to central or final waste storage. Initially, 40% of the workers understood the final disposal options and the corresponding environmental regulations. At the end of the training, 82% of the workers showed improved knowledge of treatment options and applicable rules. In addition, there was a 20% increase in the correct final waste disposal and compliance with environmental regulations, reflecting a substantial improvement in waste management.

**Figure 3:**
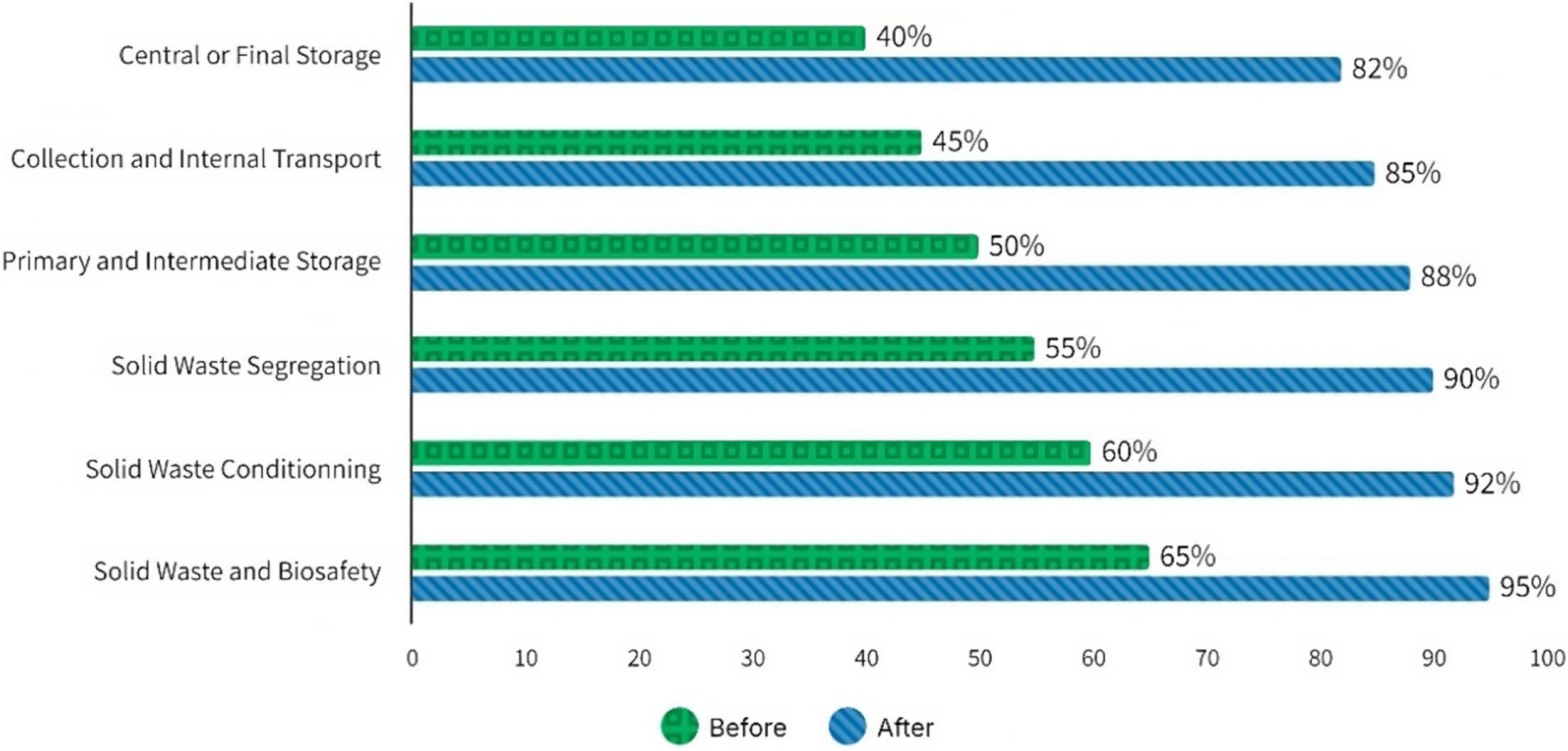
Results of the program sessions aimed at healthcare workers to improve practices and safety in handling solid waste.

The overall evaluation of the study reveals remarkable satisfaction among participants and significant improvements in waste management and biosafety. Ninety-five percent of the workers reported being satisfied or very satisfied with the training received, highlighting the program’s effectiveness. There was an overall improvement of 25% in waste management and compliance with biosafety standards, reflecting substantial progress in hospital practices. In addition, there was a 15% reduction in the risks associated with the handling and disposal of solid waste, indicating a safer and more efficient work environment. In conclusion, the training has proven effective in improving workers’ knowledge and practices, resulting in safer and more efficient management of hospital waste and reduced risks to personnel (Figure 3).

A Clinical Practice Guideline for the Prevention and Management of Occupational Exposure to Bloodborne Pathogens was implemented in 2022 based on the best available evidence from 2021. This guideline includes key indicators such as assessing adherence to prevention measures, the rate of staff training, and the awareness of the risks associated with these pathogens. In particular, the percentage of employees trained on the risks and preventive strategies related to blood and body fluids exposure is monitored.

In 2022, significant progress was achieved in implementing the Clinical Practice Guideline for the Prevention and Management of Occupational Exposure to Bloodborne Pathogens. During this year, the staff training and awareness rate reached 88.67%, reflecting an effective initial effort in training and sensitization. In addition, the safe handling rate of hazardous waste reached 90%, consolidating good practice in the segregation and disposal of potentially contaminated waste from the outset.

In 2023, a significant improvement in both indicators was observed. The staff training and awareness rate increased to 91% due to intensified training and awareness programs on the risks associated with bloodborne pathogens. The rate of safe hazardous waste management increased to 93%, improving the efficiency of hazardous waste management and reducing biohazards in the workplace.

As of August 2024, progress has continued. The staff training and awareness rate reached 94%, demonstrating an increasingly knowledgeable and committed working environment. At the same time, the rate of safe handling of hazardous waste reached 96%, demonstrating increasingly rigorous and effective control of contaminated waste management, contributing to a safer and more biohazard-protected environment.

### 3.5 Hospital-acquired infections at the private clinic

Implementing the hospital solid waste management plan at the private clinic shows a direct correlation between the correct disposal of waste and the reduction of nosocomial infections (Table 5).

**Table 5:** List of events subject to mandatory epidemiological surveillance (10).

N°	IAAS	Risk factor	UPSS
1	Bloodstream infection (primary bacteremia and clinical sepsis)	Central Venous Catheter (CVC)	Intensive Care Unit (ICU) Adult, Pediatric ICU, Neonatal ICU, Neonatology
		Peripheral Venous Catheter (CVP)	Neonatal ICU and Neonatology
		Total Parenteral Nutrition (TPN)	Adult ICU, Pediatric ICU, Neonatal ICU, Neonatal ICU
		Hemodialysis Catheter	Adult ICU
2	Urinary tract infection	Indwelling urinary catheter (PUC)	Adult ICU, Pediatric ICU, Adult and Pediatric Medicine and Surgery
3	Pneumonia	Mechanical Ventilator (MV)	Adult ICU, Pediatric ICU, Neonatal ICU
4	Surgical site infection: superficial and deep	Laparotomy cholecystectomy	Adult and Pediatric Surgery
		Cholecystectomy with laparoscopy	
		Inguinal hernioplasty (IH)	
		Hip prosthesis	Traumatology
		Cesarean Delivery (CD)	Obstetrics and Gynecology
5	Puerperal endometritis	Cesarean Birth (CP)	Obstetrics and Gynecology
		Vaginal delivery (PV)	
6	Occupational exposure to blood-borne infectious agents in health care workers	Sharps and splashing accidents	All services

Statistics of intrahospital infections from occupational exposure to blood borne infections agents in healthcare personnel (10).

**Figure 4:**
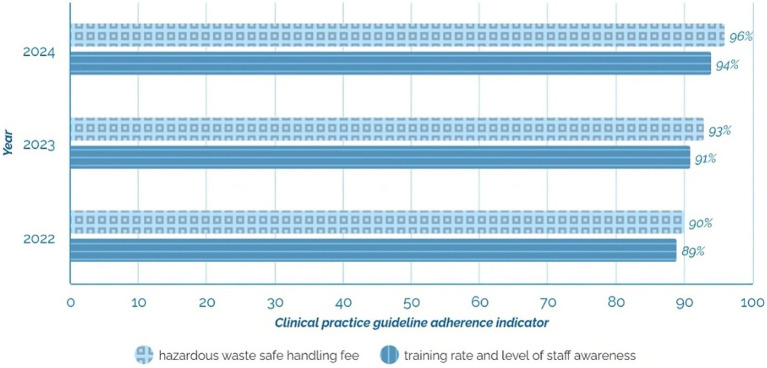
The trend in staff training rate and safe hazardous waste management rate at a private clinic (2022–2024).

**Figure 5:**
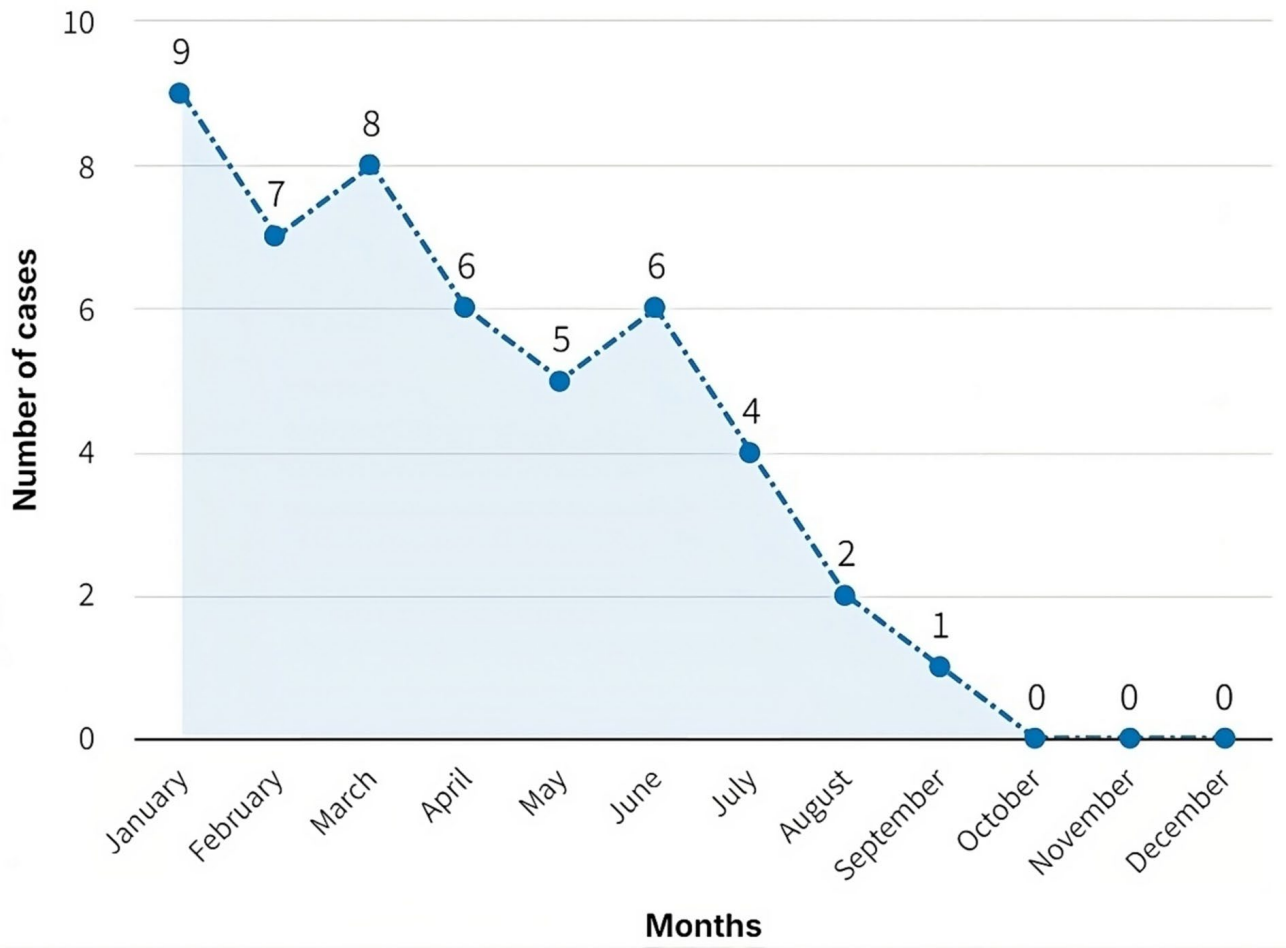
In-hospital infections in private clinic personnel due to occupational exposure to bloodborne pathogens (January–December 2021).

During the first 7 months of 2021, private clinic faced many nosocomial infections linked to poor solid waste management. Infection records showed nine cases in January, seven in February, eight in March, six in April, five in May, six in June, and four in July (see Figure 4). The high incidence of infections in these months can be attributed to the lack of training and implementation of proper waste management practices (Figure 5).

**Figure 6:**
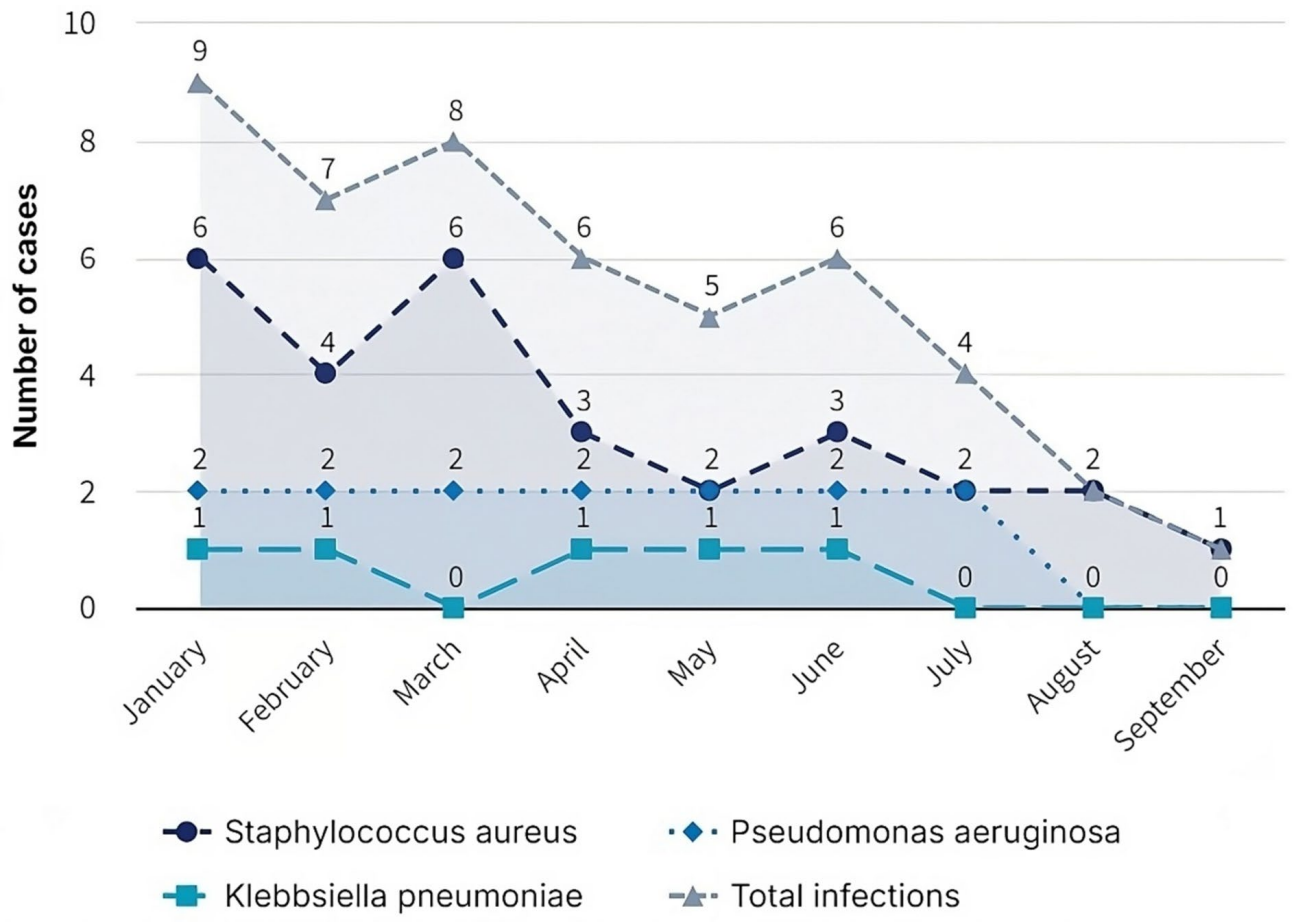
Internal report Epidemiology Area of the private clinic: Infections are broken down by type of bacteria and month.

The table presents the frequency of infections caused by three common bacterial pathogens: *Staphylococcus aureus*, *Pseudomonas aeruginosa*, and Klebsiella pneumonia over 7 months. Each month, the number of infections and the distribution of infections by each of the three microorganisms mentioned are reported (Figure 6).

Since the start of fieldwork and the implementation of a training and awareness program in August, there has been a notable decrease in hospital-acquired infections: two cases in August, one in September, and no cases were reported in the last 3 months of the year. These data show the positive and significant effect of the training plan and proper solid waste management.

The reduction in the number of nosocomial infections since August highlights the importance of continuous training and the correct implementation of efficient solid waste management practices.

At each stage, clinic staff demonstrated acceptable compliance with procedures, contributing to the reduction of infections. The training improved the staff’s understanding of waste management and fostered a culture of safety and prevention.

**Figure 7:**
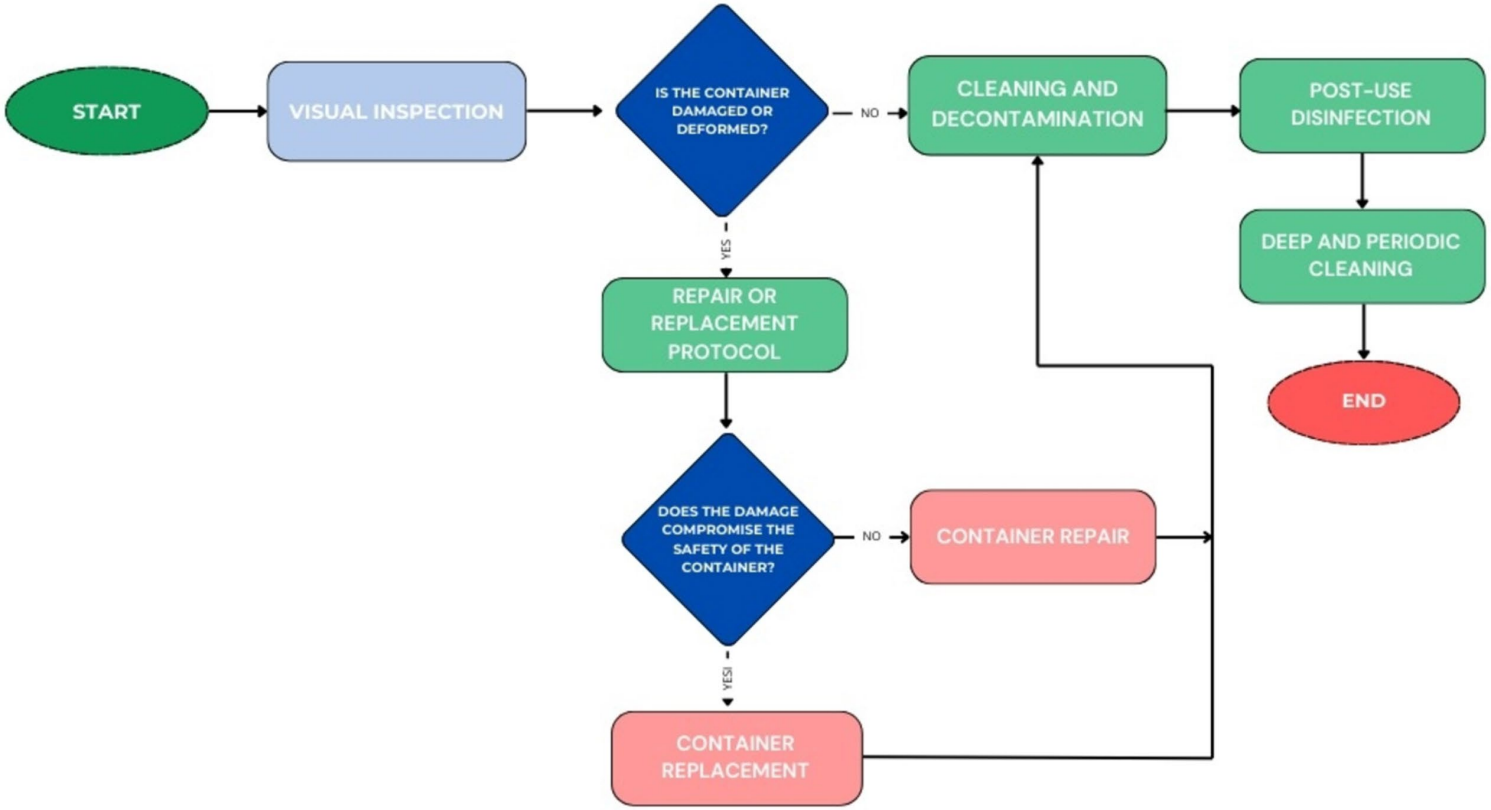
Workflow for Container Management.

The clinic has rigorous procedures for inspecting and cleaning containers used to handle potentially hazardous materials. These ensure the structural integrity of the containers through periodic inspections and thorough disinfection practices to minimize the risk of leakage or exposure to pathogens. The protocols also provide for the immediate repair or replacement of damaged containers, reinforcing preventive measures. These procedures follow the scheme shown in Figure 7.

**Figure 8:**
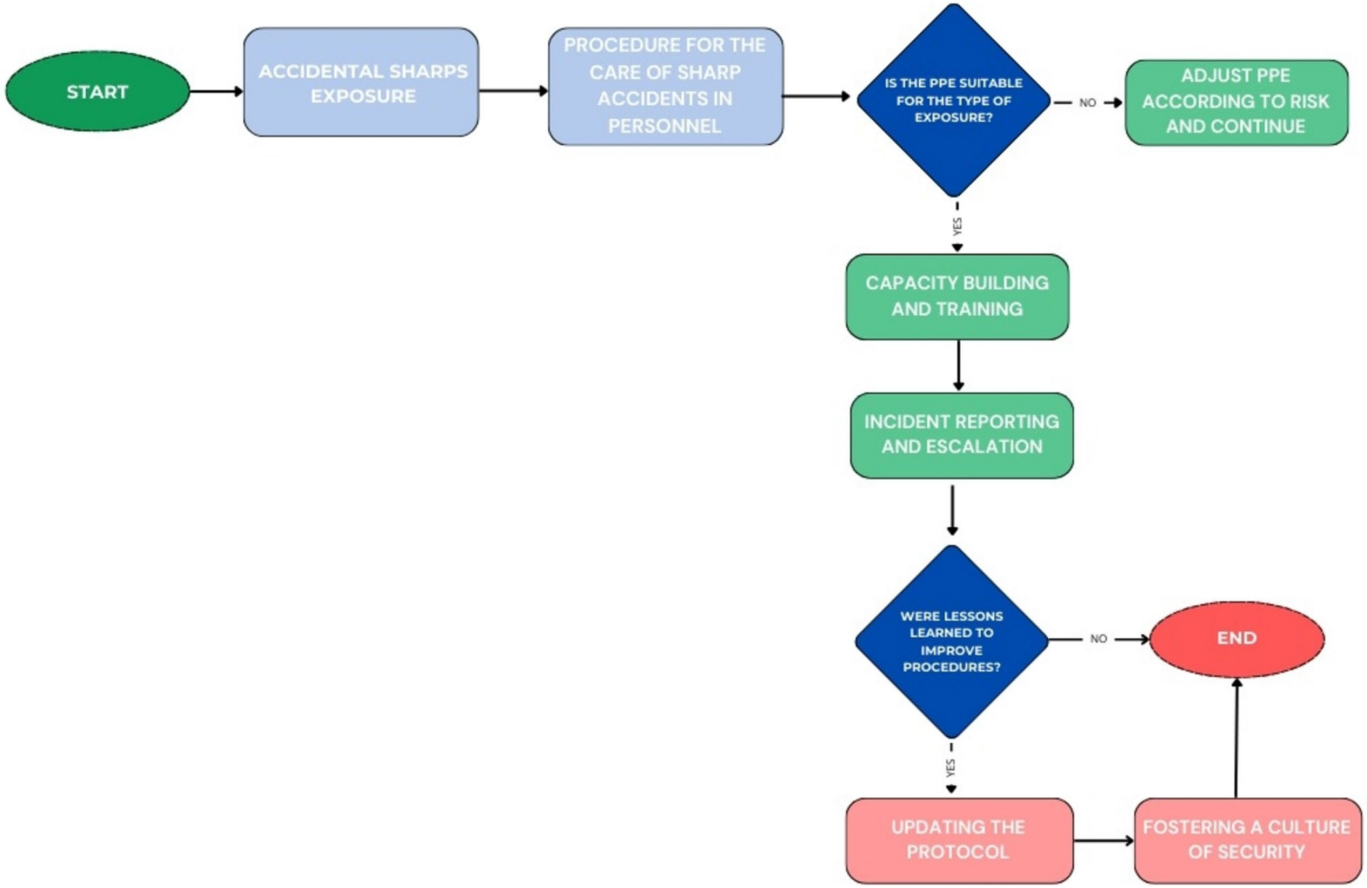
Flow Chart for Management of Accidental Sharps Exposures.

Regarding incidents involving contaminated sharps, the clinic has an immediate response protocol that includes administering first aid, appropriately using personal protective equipment (PPE), and mandatory staff training in this area. This procedure, complemented by a structured incident reporting system through an institutional platform, allows for detailed analysis of events and fosters the continuous improvement of safety policies (see Figure 8).

Comparison of infection data before and after the intervention demonstrates the effectiveness of a comprehensive approach to waste management. Clinics can use this experience as a model to optimize their waste management and provide staff with a safer environment.

Thus, the private clinics have demonstrated that efficient hospital solid waste management, supported by a comprehensive training plan, is critical to reducing the risk of nosocomial infections, improving occupational health and safety, and promoting a safer and healthier hospital environment.

Since the implementation of the Clinical Practice Guideline for the Prevention and Management of Occupational Exposure to Bloodborne Pathogens, there has been a steady improvement in both the rate of staff training and their level of awareness of the risks associated with bloodborne pathogens. Similarly, the rate of safe management of hazardous waste has shown significant progress over the years, reinforcing safety and prevention measures in work environments. Another relevant indicator is the rate of safe handling of hazardous waste, which measures the effectiveness of practices for handling and disposing of potentially contaminated waste, such as biological waste generated in healthcare settings.

**Figure 9:**
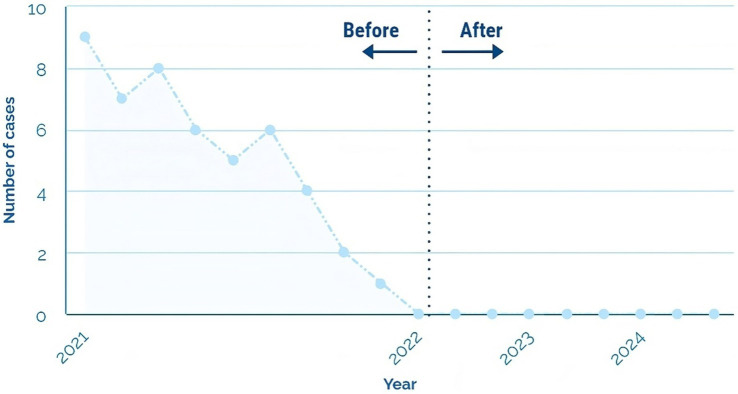
We monitor nosocomial infections in private clinic staff due to occupational exposure to bloodborne pathogens (2021–2024).

During 2022 and until August 2024, this management tool has significantly contributed to the continuous improvement of patient care by applying sound scientific evidence. In addition, it has promoted a safer and more efficient working environment in biohazard management (Figure 9).

## 4 Discussion

The analysis of solid waste management at the private clinic has revealed significant results in reducing intrahospital infections, highlighting the direct relationship between staff training and proper waste management (2, 7). It was estimated that the daily generation of biocontaminated waste was 232.76 kilograms, which allows determining that successful hospital waste management will largely depend on the actual waste estimation (8); hence, it is necessary to implement rigorous protocols in high-risk areas. This figure aligns with the findings of Balladares (2, 3), who emphasizes that effective municipal environmental management is essential to mitigate the risks associated with improper waste management in hospital settings. The study also evidences that floors 1 and 2 were the main generators of biocontaminated waste, suggesting the need to focus interventions in these critical areas.

The conditioning stage, which involved preparing areas with suitable materials such as bags and rigid containers, facilitated proper separation from the point of generation. This step was crucial to ensure that hazardous waste was handled correctly, minimizing the time staff and patients were exposed to infectious agents. Studies have shown that correctly preparing work areas is a determining factor in reducing hazards associated with hospital waste management (2, 11).

Acceptable compliance with the established guidelines regarding segregation and initial storage was observed. The staff showed sufficient knowledge about the correct waste classification, an aspect essential to preventing cross-contamination and ensuring safety in patient care (2, 6, 7). Correct segregation decreases the risk of infections and optimizes waste management, as supported by studies on continuous training in solid waste management (2, 5–7).

Internal waste transport, performed by properly equipped personnel and using appropriate vehicles, ensured safe and efficient waste collection and avoided cross-contamination. Several studies have highlighted the importance of safe and well-managed internal transport, which is vital to prevent the spread of pathogens within healthcare facilities (2, 5–7, 9, 12).

Finally, in the final storage phase, the waste was temporarily deposited in suitable environments before treatment or final disposal, which ensured safe and orderly handling. Proper storage is crucial to maintaining hygiene and safety in hospital establishments, as indicated in the World Health Organization guidelines on the secure management of hospital waste (2, 5–7, 9, 12).

After implementing the Hospital Solid Waste Management Plan, the reduction of in-hospital infections as of August 2021 evidences a positive change in the clinic’s public health. In the first 7 months of the year, a high incidence of infections was observed, with a total of 43 cases, which coincides with the findings of Linares and Ortega (8) on the link between inadequate waste management and increased infections in hospital settings. The drastic decrease in the number of infections, which reached zero cases in the months from October to December, highlights the positive impact of the training implemented, corroborating the findings of studies that have shown that lack of knowledge in waste management is a critical factor in the occurrence of nosocomial infections (2, 3, 5–9, 12).

These results underscore the training plan’s effectiveness and highlight the need for rigorous management in high-risk areas where the production of biocontaminated and particular waste is significant. It is recommended that healthcare personnel commit to proper waste management practices by actively participating in ongoing training to maintain optimal biosafety standards. This need is supported by studies emphasizing that constant personnel training is critical to facing waste management challenges in the hospital context (2, 3, 5–9, 12).

In conclusion, the implementation of a training plan to prepare personnel in solid waste management at the private clinic has not only led to better waste management but has also directly influenced the reduction of nosocomial infections. This study provides solid evidence of the need to train personnel in waste management, which translates into improvements in public health and patient safety. Future research should continue to explore the effectiveness of similar interventions in other healthcare settings to strengthen the link between waste management and public health.

### 4.1 Recent developments in occupational safety

In recent years, significant improvements in occupational safety regulations and practices have been introduced. Globally, the most recent guidelines emphasize ongoing staff training and the use of innovative technologies to reduce the risks associated with hospital waste. For example, the use of automated segregation systems and containers with antimicrobial technology is gaining popularity as a strategy to mitigate exposure to infectious agents (13).

### 4.2 Socioeconomic and policy implications

Hospital waste management also has profound socioeconomic implications. Local communities near waste disposal sites often experience increased health problems related to environmental contamination, exacerbating social inequality. Implementation of integrated strategies, such as recycling programs and efficient wastewater management, could reduce these impacts and promote community well-being (5). In addition, public policies that integrate occupational safety into local and regional planning have proven to be effective in addressing these issues (8).

## 5 Conclusion

Implementing a comprehensive hospital solid waste management plan and ongoing staff training at the private clinic during 2021 have proven effective strategies for reducing nosocomial infections. The revealing decrease in the number of infections from August confirms the effectiveness of training in properly managing solid waste.

The production and management of biocontaminated and waste areas highlighted the need for rigorous management in high-risk areas. Proper segregation, transport, and storage of these wastes are essential to reducing infection risks and ensuring a safe environment for staff and patients.

Adherence to solid waste management protocols was assessed as acceptable at all stages, supporting the effectiveness of the practices implemented. These findings underscore the importance of maintaining and reinforcing biosafety practices through ongoing healthcare personnel training and engagement.

### 5.1 Recommendations

It is recommended that all personnel involved be fully committed to proper solid waste management practices. Active participation in training and strict adherence to protocols are essential to maintain high biosafety standards.

Maintaining an ongoing training program for all persons involved in waste management is crucial. These trainings should be updated regularly to incorporate new trends and efficient practices in hospital waste management.

Periodic waste management evaluations are suggested to ensure compliance with protocols and detect areas for improvement. On-site visits can be an effective tool for monitoring personnel behavior and correctly implementing safety measures.

The clinic should ensure the availability of adequate supplies and equipment for waste management, such as bags, rigid containers, and specialized vehicles for internal transport. Investment in these resources is essential for safely and efficiently managing biocontaminated and waste.

It is recommended that a culture of safety and prevention be fostered among all clinic personnel. Creating a work environment prioritizing safety and health can significantly reduce the risks associated with hospital solid waste.

### 5.2 Limitations

Limitations of this research include restricting the generalizability of the results because it was conducted in a single clinic, which may not reflect the diversity of practices in other institutions. In addition, reliance on self-reporting to assess protocol compliance may introduce bias. The limited duration of post-training follow-up may not capture long-term effects on infection reduction.

## Data Availability

The datasets presented in this article are not readily available because these data were authorized by the private clinic for the purpose of this research. Requests to access the datasets should be directed to Jorge Ibáñez, jorge_23_5@outlook.com.

## Footnotes

^1^This work is the result of the Thesis carried out by Augusto Jorge Antonio Ibáñez-Cruz, with updates and contributions made by the authors of this research (2).
